# Mesenchymal Stem Cells Loaded with p5, Derived from CDK5 Activator p35, Inhibit Calcium-Induced CDK5 Activation in Endothelial Cells

**DOI:** 10.1155/2016/2165462

**Published:** 2016-08-29

**Authors:** Wen-Hui Fang, Shant Kumar, Garry McDowell, David Smith, Jurek Krupinski, Peter Olah, Raid Saleem Al-Baradie, Mohammad Othman Al-Rukban, Eugene Bogdan Petcu, Mark Slevin

**Affiliations:** ^1^School of Healthcare Science, Manchester Metropolitan University, Manchester, UK; ^2^Department of Neurology, Hospital Universitari Mútua de Terrassa, Terrassa, Barcelona, Spain; ^3^University of Medicine and Pharmacy, Tirgu Mures, Romania; ^4^Department of Medical Laboratories, College of Applied Medical Sciences, Majmaah University, Al Majma'ah, Saudi Arabia; ^5^Griffith University School of Medicine and Queensland Eye Institute, Griffith University, Nathan, QLD, Australia

## Abstract

The potential use of stem cells as therapeutics in disease has gained momentum over the last few years and recently phase-I clinical trials have shown favourable results in treatment of a small cohort of acute stroke patients. Similarly, they have been used in preclinical models drug-loaded for the effective treatment of solid tumours. Here we have characterized uptake and release of a novel p5-cyclin-dependent kinase 5 (CDK5) inhibitory peptide by mesenchymal stem cells and showed release levels capable of blocking aberrant cyclin-dependent kinase 5 (CDK5) signaling pathways, through phosphorylation of cyclin-dependent kinase 5 (CDK5) and p53. These pathways represent the major acute mechanism stimulating apoptosis after stroke and hence its modulation could benefit patient recovery. This work indicates a potential use for drug-loaded stem cells as delivery vehicles for stroke therapeutics and in addition as anticancer receptacles particularly, if a targeting and/or holding mechanism can be defined.

## 1. Introduction

Despite all recent advances in ischemic stroke research, treatments and recovery rates of patients have not been improved significantly [1]. Such a clinical scenario underscores the importance of investing in new therapeutic approaches. The preclinical and clinical studies have shown that stem cell-based therapies have the enormous potential for the treatment of a wide range of diseases [2]. Adult stem cells have been studied extensively and are already a successful source of FDA-approved treatments for a number of diseases including juvenile diabetes and Parkinson's disease. In contrast to the treatment of diseases such as diabetes and Parkinson's disease where restricted populations are lost, multiple cell types are lost in stroke and it will be important to repair both blood vessels (endothelial cells, smooth muscle cells, and pericytes) and neurons and glial cells [3–5]. Another important distinction is that stroke is an acute injury limited in time, which might make the brain more hospitable to transplantation than in other diseases. Mesenchymal stem cells (MSCs, also called mesenchymal stromal cells) have the advantage of being relatively easy to propagate* in vitro* and implantation of autologous MSCs into patients has fewer ethical problems and is not subject to alloimmunization and thus may represent an ideal candidate for cellular therapies [6].

Cyclin-dependent kinase 5 (CDK5), a serine/threonine kinase, in complex with its activators, p35 (protein of 35 kDa) and p39 (protein of 39 kDa), is essential for early neurodevelopment in mammals [1]. However, a variety of neurotoxic conditions, such as ischemic brain damage, oxidative stress, amyloid *β* peptide (A*β*), excitotoxicity, calcium dyshomeostasis, and inflammation, induce influx of calcium ions and a rise in the intracellular Ca^2+^, thereby promoting activation of calpain, a Ca^2+^-activated protease, which in turn cleaves p35 into p25 and a p10 fragment [7, 8]. p25 forms a more stable CDK5-p25 hyperactive complex, which causes aberrant hyperphosphorylation of various substrates of CDK5 like Tau and neurofilament, leads to neuronal apoptosis, and is associated with neuropathology. Therefore, a therapeutic approach directed specifically at CDK5-p25 complex might prove successful. However, kinase inhibitors (such as roscovitine) are not very specific as they inhibit not only CDK5-p25 but also CDK5-p35 and other CDKs, leading to serious side effects and thereby reducing the therapeutic efficacy. In order to overcome this problem, several peptides consisting of amino acid residues of p35, such as CDK5 inhibitory peptide (CIP, a peptide of 125 amino acid residues), p10, and p5 have been generated and proven to specifically reduce CDK5-p25 increased activity without affecting the normal endogenous CDK5-p35 or other CDKs activities [9–15]. In particular, p5 also reduced neuronal apoptosis induced by hypoxia/ischemia brain injury, high glucose-mediated toxicity, or A*β* stress [12–14]. Based on these encouraging findings, we decided to investigate whether MSCs could uptake and release p5 peptide and then inhibit CDK5 activation induced by calpain in endothelial cells as well as the potential of MSCs as drug carrier to deliver p5 to the peri-infarcted regions and protect endothelial cells and neurons against CDK5-p25 induced toxicity in these regions.

## 2. Materials and Methods

### 2.1. Cell Culture

Human adipose-derived mesenchymal stem cells (hADMSCs) were kindly provided by Professor Giulio Alessandri and Professor Valentina Cocce. HAD-MSCs were isolated from periumbilical fat tissue and characterized as described [16]. Cells were grown in stem cells medium (SCM) comprised of 80% Iscove's modified Dulbecco's medium (IMDM; Sigma-Aldrich) containing 5% fetal bovine serum (FBS; Sigma-Aldrich), 10% NeuroCult medium (Stem Cell Technologies), and 10% endothelial basal medium (EBM) (Lonza) in a humidified incubator with 5% CO_2_ at 37°C. Bovine aortic endothelial cells (BAECs) were cultured in Dulbecco's Modified Eagles Medium (DMEM; Lonza) supplemented with 10% FBS.

### 2.2. p5 Priming of hADMSCs

p5, a 24-residue peptide, derived from p35, the CDK5 activator, was chemically synthesized and the single biotin moiety was conjugated on the N-terminus [15] (21st Century Biochemicals). The sequence of p5-biotin peptide is Biot-Ahx-KEAFWDRCLSVINLMSSKMLQINA-OH. The toxicity of p5 on hADMSCs was determined in a 24-hour alamarBlue assay (cytotoxicity test; Life Technologies) and in a 3-day alamarBlue assay (antiproliferative test). Based on these results, the p5 priming of hADMSCs was carried out with three doubling concentrations, 3, 6, and 12 *μ*g/mL. Briefly, subconfluent cultures (2 × 10^5^) of hADMSCs were exposed to different dosages of p5. After 24-hour of incubation, the cells were washed twice with PBS and trypsinized. Cells were then seeded in a new flask in fresh SCM. After 24-hour of culture, the cell conditioned medium (CM) was collected and tested for protective effects* in vitro* on BAECs. CM from untreated hADMSCs cultured under the same conditions was used as control.

### 2.3. Immunofluorescence Analysis

2 × 10^4^ hADMSCs plated on glass coverslips in 24-well multiwell plates in SCM were grown to subconfluence over 24 hours. The cells were then treated with 3 *μ*g/mL p5. At the end of the specified incubation period (1, 2, 4, 8, or 24 hours), the cells were fixed in 4% paraformaldehyde. For other time points, the cell media containing p5 were removed after 24-hour incubation, and cells were washed 3 times in PBS and fed with fresh SCM. At the corresponding time point, the cells were fixed in 4% paraformaldehyde.

BAECs (1 × 10^4^) plated on glass coverslips in 24-well multiwell plates in DMEM were grown to subconfluence over 24 hours. The cells were treated with different concentrations of p5 or CM of p5-primed hADMSCs for 24 hours. Cells were fixed in 4% paraformaldehyde.

p5 was detected using anti-fluorescein (biotin) antibody (Abcam) 1 : 200 in milk. Coverslips were then mounted using Vectashield mounting medium with DAPI (Vector Laboratories) and sealed with nail varnish. Images were obtained at 200x magnification using Imager Z1 fluorescence microscope (Zeiss) and fluorescence intensity was quantified by ImageJ 1.49 software (https://imagej.nih.gov/). Presented values are averages of six images.

### 2.4. Western Blot Analysis

7 × 10^4^ BAECs plated in 6-well multiwell plates in DMEM were grown to subconfluence over 24 hours. The cells were treated with 5 *μ*M calcium ionophore A23187 (Sigma-Aldrich) and 2.5 mM CaCl_2_ (Sigma-Aldrich) with or without p5 or CM of p5-primed hADMSCs. The cells were lysed in RIPA buffer containing protease inhibitor cocktail and phosphatase inhibitor cocktail (Sigma-Aldrich). Western blot was performed using 10% SDS-PAGE with 40 *μ*g total proteins. The proteins were transferred to polyvinylidene difluoride (PVDF) membrane. The membrane was blocked in a blocking solution (pH 7.4) composed of 1% BSA (Sigma-Aldrich) dissolved in TBS containing 0.1% Tween-20 (TBS-Tween, pH 7.4) for 1 hour and incubated with the primary antibodies including anti-phospho-CDK5 (pTyr^15^) (Sigma-Aldrich, 1 : 500), anti-CDK5 (Cell Signaling Technology, 1 : 1,000), anti-p35 (Abcam, 1 : 100), anti-phospho-ERK1/2 (Cell Signaling Technology, 1 : 2,000), anti-ERK1/2 (Cell Signaling Technology, 1 : 1,000), anti-phospho-p53 (pSer^15^) (Cell Signaling Technology, 1 : 500), anti-p53 (Sigma-Aldrich, 1 : 500), anti-active caspase-3 (Sigma-Aldrich, 1 : 200), anti-procaspase-3 (Abcam, 1 : 250), and anti-*α*-tubulin (Abcam, 1 : 5,000). The horseradish peroxidase (HRP) conjugated secondary antibodies, goat anti-rabbit IgG or goat anti-mouse IgG (Daco), were used. The immunoreactivity was visualized with chemiluminescence detection kit (Pierce ECL Western Blotting Substrate; Thermo Scientific). Western blot images were captured with Chemidoc Touch Imaging system (Bio-Rad). Molecular weights of proteins were estimated by comparison with MagicMark XP western protein standard (Invitrogen). Blots shown are one of at least two independent experiments performed.

### 2.5. LC-MS Analysis

Liquid chromatography-mass spectrometry (LC-MS) analysis was performed using an Agilent 1290 LC system coupled to an Agilent 6530 high definition quadrupole time-of-flight (q-TOF) mass spectrometer, equipped with an Agilent Jet Stream technology dual electrospray source. The p5 peptides were prepared in methanol (OPTIMA LC-MS grade; Fisher Scientific) and chromatographic separation was performed on Waters Symmetry C18 column (2.1 × 50 mm i.d., 3.5 *μ*m; Agilent Technologies). The column was eluted at a flow rate of 0.5 mL/minutes using water as mobile phase A and acetonitrile as mobile phase B, both containing 0.1% formic acid. 0.1% of mobile phase B was kept constant and then a linear gradient was started to reach 100% mobile phase B in 15 minutes and held until 20 minutes; then gradient is set to reach 50% B in 25 minutes and then held till 40 minutes; final gradient is set to reach 0.1% B in 55 minutes and then held at 0.1% B till 60 minutes. The column temperature was maintained at 20°C and injection volume was 10 *μ*L.

The column effluent was directly introduced into the electrospray source of q-TOF mass spectrometer. Analyses were performed in the positive ion mode with scan range of 100–3200 *m*/*z* and scan rate of 1 spectra per second. The electrospray source conditions were as follows: capillary 2000 V, nozzle 500 V, fragmentor 80 V, skimmer 45 V, drying gas 80°C at 8 L/minutes, nebulisation gas 15 psig, and sheath gas 350°C at 10 L/minutes. Agilent Mass Hunter software was used to acquire and process the data (acquisition version B.05.00 and qualitative data analysis version B.05.00).

### 2.6. *In Vitro* Proliferative Assay

The protective effect of both p5 or CM of p5 primed hADMSCs on BAECs proliferation after the treatment of Ca^2+^ ionophore combined with CaCl_2_ was determined by the alamarBlue assay (Life Technologies). Briefly, 1.2 × 10^3^ BAECs were plated in 96-well multiwell plates in DMEM. The cells were then treated with 5 *μ*M calcium ionophore A23187 (Sigma-Aldrich) and 2.5 mM CaCl_2_ (Sigma-Aldrich) with or without p5 or CM of p5-primed hADMSCs. Three wells were devoted for the assay every day for 3 days, and time-course of cell proliferation was monitored. In practice, 10 *μ*L of the alamarBlue reagent (Life Technologies) was added to each well and the plates were incubated at 37°C for 4 hours in a humidified atmosphere with 5% CO_2_ before recording fluorescence (530_Ex_/590_Em_) using a Synergy HT multidetection microplate reader (BioTek Instruments). According to the standard curve for BAECs generated from fluorescence signals plotted against the number of cells (between 0 and 2.0 × 10^4^ BAECs), cell numbers were calculated from the fluorescence signals.

## 3. Results

### 3.1. Uptake and Release of p5 by hADMSCs

p5 is a 24-residue peptide derived from p35, the CDK5 activator. Since p5 is the basic peptide and needs acetic acid to be fully dissolved, we tested the toxicity of the p5 solution on both hADMSCs and BAECs. The maximum dose of p5, which will not affect cell viability and proliferation, was 12 *μ*g/mL using alamarBlue assay. Based on these results, three doubling concentrations, 3, 6, and 12 *μ*g/mL, were employed in the current study.

**Figure 1:**
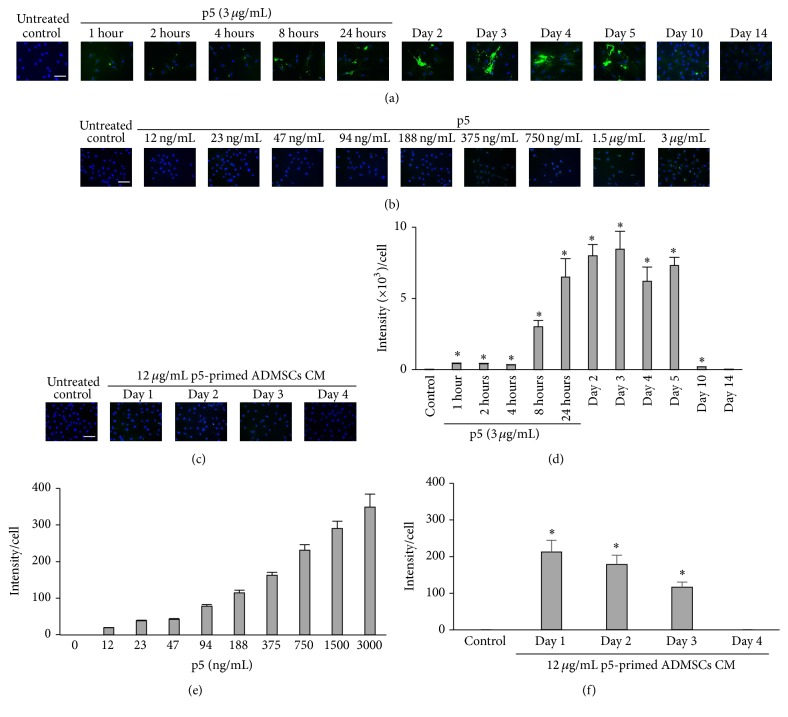
The p5 internalization and release by hADMSCs. (a) The internalization of p5 was analyzed by fluorescence microscopy in hADMSCs primed 1, 2, 4, 8, or 24 hours with 3 *μ*g/mL p5 (green). Cells were also observed after 24 hours priming on Days 2, 3, 4, 5, 10, and 14. (b) The internalization of p5 was analyzed by fluorescence microscopy in BAECs primed with different doses of p5 (green) for 24 hours. The intensity of p5 staining in cytoplasm was correlated with the dosages of p5. (c) Subconfluent cultures (2 × 10^5^) of adherent hADMSCs were exposed to 12 *μ*g/mL biotinylated p5 for 24 hours. After several washes and trypsinization, p5-primed hADMSCs were further cultured and their conditioned medium (CM) was collected every 24 hours. The internalization of p5 was analyzed by fluorescence microscopy in BAECs treated with p5-primed hADMSCs CM for 24 hours. Scale bar: 100 *μ*m. (d) The fluorescein staining intensities of p5 in hADMSCs in (a) were quantified in the bar graph as means ± SD (error bars). Significance (denoted as *∗*) was calculated compared to the untreated control group using Student's *t*-test (with *p* < 0.01, *n* = 6). (e) The fluorescein staining intensities of p5 in BAECs in (b) were quantified in the bar graph as means ± SD (error bars). (f) The fluorescein staining intensities of p5 in BAECs in (c) were quantified in the bar graph as means ± SD (error bars). Significance (denoted as *∗*) was calculated compared to the untreated control group using Student's *t*-test (with *p* < 0.01, *n* = 6).

Subconfluent culture (2 × 10^4^) of adherent hADMSCs was exposed to 3 *μ*g/mL biotinylated p5. The internalization of p5 into hADMSCs was investigated by fluorescence microscopy using anti-fluorescein (biotin) antibody over time. After 1 hour of priming, the internalization of p5 by hADMSCs was appreciable (Figures 1(a) and 1(d)). The staining was gradually intensified and enriched in cytoplasm at the end of priming (24 hours). After 24 hours, the distribution of p5 remained in cytoplasm for 5 days whereas the staining decreased from day 10, suggesting its possible excretion, degradation, or both.

To assess if p5-primed hADMSCs could release p5 into the media, subconfluent culture (2 × 10^5^) of adherent hADMSCs was exposed to 12 *μ*g/mL biotinylated p5 for 24 hours. After several washes and trypsinization, p5-primed hADMSCs were further cultured and their conditioned medium (CM) was collected every 24 hours and tested on BAECs. There was evident p5 staining in BAECs treated with CM of p5-primed hADMSCs collected on Days 1 and 2, the staining decreased on Day 3, and no staining was observed from Day 4 onwards, suggesting that p5-primed hADMSCs continuously released p5 into medium for 3 days, with the peak release within the first 48 hours (Figures 1(c) and 1(f)). Comparing the p5 staining intensity in BAECs treated with pure p5 and CM, we estimated that the p5 in the CM collected on Days 1 and 2 was equivalent to 212.60 ± 31.89 ng/mL and 178.41 ± 24.98 ng/mL, respectively, and the p5 in the CM collected on Day 3 was equivalent to 116.50 ± 13.98 ng/mL (Figures 1(b), 1(c), 1(e), and 1(f)).

### 3.2. LC-MS Analysis of p5

**Figure 2:**
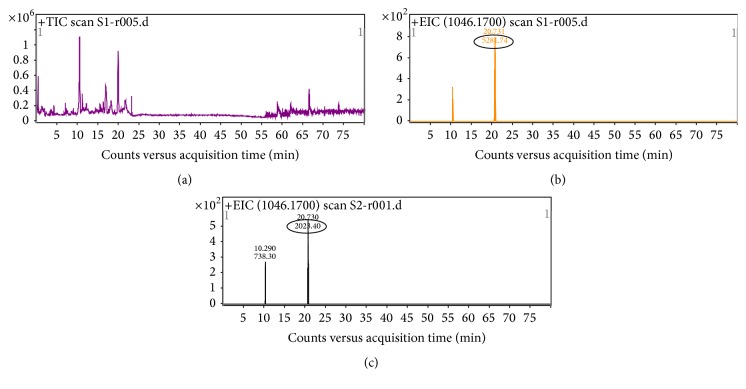
LC/MS with Q-TOF analysis of p5. (a) The total ion chromatogram (TIC) of 200 ng/mL p5. (b) Positive ESI mass spectra (1046.1700 *m*/*z* corresponding to [M]^3+^ in excellent agreement with the expected value) of 20.70 ± 0.05 minutes of 200 ng/mL p5. The area of peak was circled. The average peak area for 200 ng/mL p5 from 6 tests was 5563.63 counts. (c) Positive ESI mass spectra (1046.1700 *m*/*z*) of 20.70 ± 0.05 minutes of 100 ng/mL p5. The area of peak was circled. The average peak area for 100 ng/mL p5 from 6 tests was 2430.55 counts. The ESI-MS spectra showed no peak for 50 ng/mL p5 (data not shown).

In order to quantify the p5 release by p5-primed hADMSCs, we tested p5 by LC-MS analysis. LC-MS analysis can detect p5 and the measured ion 1046.1700 *m*/*z* corresponding to [M]^3+^ that is in excellent agreement with the expected value (Figure 2). The average peak area for 200 ng/mL p5 from 6 tests was 5563.63 counts and 2430.55 counts for 100 ng/mL p5 (Figures 2(b) and 1(c)). The ESI-MS spectra showed no peak for 50 ng/mL p5. The results indicated that LC-MS analysis can quantitate p5, which was found to be linear between 100 and 200 ng/mL among the range of released p5 by p5-primed hADMSCs within the first 3 days estimated by immunofluorescence assay (Figures 1(c) and 1(f)), but undetectable at 50 ng/mL. LC-MS analysis will be used for the quantification of the p5 release by p5-primed hADMSCs in the future work.

### 3.3. The Activation of CDK5 by Calpain in Cultured Endothelial Cells

**Figure 3:**
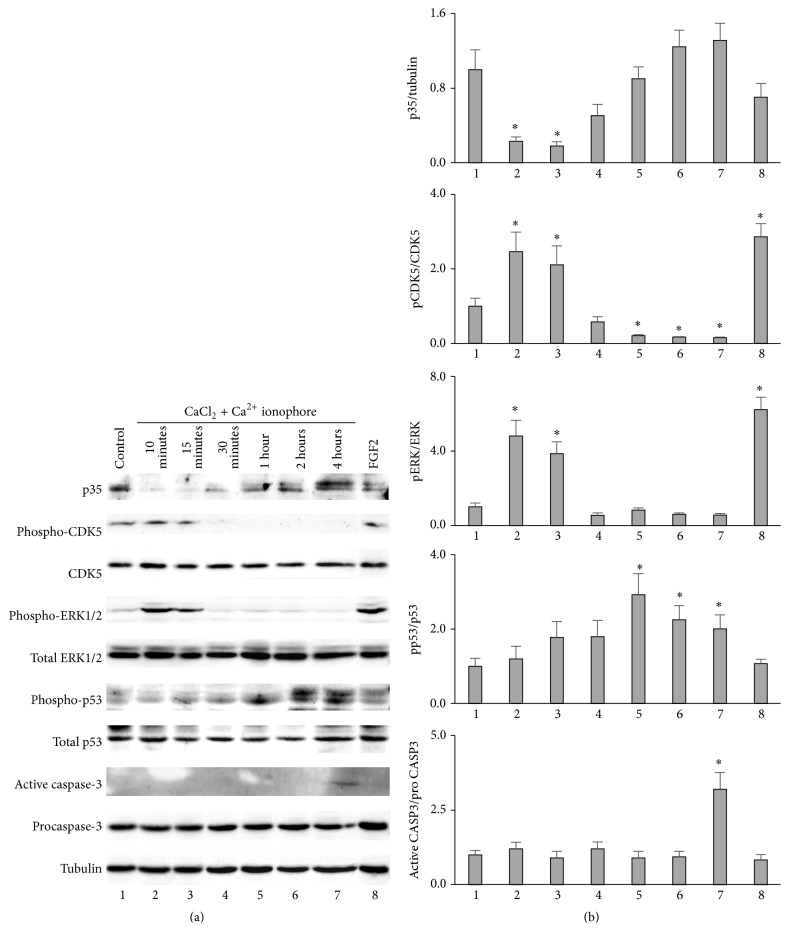
The activation of CDK5 by calpain in cultured BAECs. (a) The cultured BAECs were treated with 5 *μ*M calcium ionophore A23187 and 2.5 mM CaCl_2_ for 0, 10 minutes, 15 minutes, 30 minutes, 1 hour, 2 hours, or 4 hours. At the end of the treatment, the cells were collected, suspended in SDS-PAGE sample buffer, and boiled for 5 minutes. Each sample was run on a 10% SDS-PAGE gel and blotted with anti-p35, anti-phospho-CDK5 (pTyr^15^), anti-phospho-ERK1/2, anti-phospho-p53 (pSer^15^), anti-active caspase-3, anti-CDK5, anti-ERK1/2, anti-p53, anti-procaspase-3, and anti-tubulin antibodies. The first lane was loaded with the extract from untreated BAECs as control. The cultured BAECs treated with 10 ng/mL FGF2 for 10 minutes were used as positive control for the phosphorylation of ERK1/2 (lane 8). Blots shown are one of at least three independent experiments performed. (b) Densitometric analysis of immunoblots quantification. Data are means ± SD (error bars). Significance (denoted as *∗*) was calculated compared to the untreated control (lane 1) using Student's *t*-test (with *p* < 0.05, *n* = 3).

A previous study had showed that Ca^2+^ ionophore alone and in combination with CaCl_2_ activated endogenous calpain, thereby activating CDK5 by the cleavage of p35 to p25 in the cultured neurons [7]. We investigated whether combined treatment of Ca^2+^ ionophore and CaCl_2_ is able to activate and phosphorylate CDK5 through calpain in cultured endothelial cells. The western blotting results showed that the levels of p35 decreased whereas the phosphorylation levels of CDK5 increased after 10 and 15 minutes of treatment of Ca^2+^ ionophore and CaCl_2_; thereafter p35 levels increased and pCDK5 levels decreased immediately, suggesting that Ca^2+^ ionophore and CaCl_2_ treatment activated endogenous calpain and transiently cleaved p35 into p25, which in turn activated and phosphorylated CDK5 in endothelial cells (Figure 3).

Because calpain was reported to be activated during apoptotic cell death, we suspected a link between the activation of CDK5 and cell death [1, 17]. Indeed, the Ca^2+^ ionophore and CaCl_2_ treatment induced cell death in endothelial cells as early as 10 minutes' treatment; we therefore checked the MAPK signaling pathway and genes involved in apoptosis [1]. The phosphorylation levels of ERK1/2 showed the similar pattern as pCDK5 whereas phospho-p53 expression increased gradually following the 10 minutes' treatment of Ca^2+^ ionophore and CaCl_2_ (Figure 3). For phospho-p53 blots, preabsorption needs to be undertaken to ensure validity of its identification since there are two bands close together. In contrast, caspase-3 was activated at 4 hours of treatment, much later than p53 (Figure 3).

### 3.4. p5-Primed hADMSCs Released p5 and Inhibited CDK5 Activation by Calpain in Cultured Endothelial Cells

**Figure 4:**
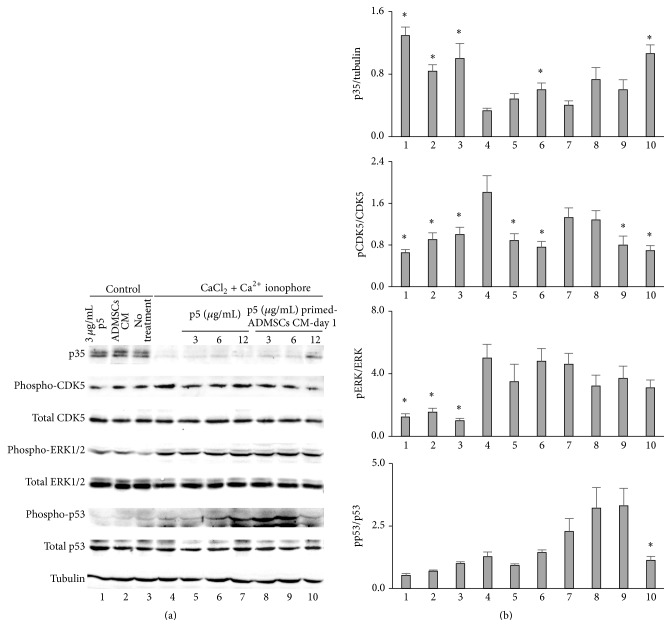
p5 and CM from p5-primed hADMSCs inhibited CDK5 and p53 activation by calpain in cultured BAECs. (a) The cultured BAECs were treated with p5 (lanes 5–7) or conditioned medium (CM) from p5-primed hADMSCs collected on Day 1 (lanes 8–10) for 1 hour and then further treated with 5 *μ*M calcium ionophore A23187 and 2.5 mM CaCl_2_ for 10 minutes. The 3 *μ*g/mL p5 treatment alone (lane 1), CM from untreated hADMSCs (lane 2) and untreated BAECs (lane 3) were used as control. The p35 cleavage and the phosphorylation of CDK5 and p53 were suppressed by p5 and CM from p5-primed hADMSCs. (b) Densitometric analysis of immunoblots quantification. Data are means ± SD (error bars). Significance (denoted as *∗*) was calculated compared to cells treated with 5 *μ*M calcium ionophore A23187 and 2.5 mM CaCl_2_ (lane 4) using Student's *t*-test (with *p* < 0.05).

We investigated whether p5 could inhibit the activation of CDK5 induced by calpain. Compared with the 10 minutes' treatment of Ca^2+^ ionophore and CaCl_2_ alone, all the three concentrations of p5 (3, 6, and 12 *μ*g/mL) suppressed the cleavage of p35 and phosphorylation of CDK5 induced by Ca^2+^ ionophore and CaCl_2_. The highest and the least inhibition was caused by 6 *μ*g/mL and 12 *μ*g/mL, respectively (Figure 4(a), lanes 4–7). More interestingly, 3 *μ*g/mL p5 prevented the activation of p53 by Ca^2+^ ionophore and CaCl_2_, suggesting p5 might protect cells from apoptosis by inhibiting p53. The less effectiveness of higher dosages of p5, especially 12 *μ*g/mL, might be due to the relative higher concentration of acetic acid in these p5 solutions. Surprisingly, pERK1/2 levels were not affected by p5 treatment, indicating that ERK1/2 was activated in endothelial cells by Ca^2+^ ionophore and CaCl_2_ treatment independently of CDK5 signaling.

We then investigated whether hADMSCs primed with p5 would release p5 in the sufficient amount to inhibit the p35 cleavage and CDK5 activation induced by calpain. The hADMSCs were exposed to three dosages of p5 (3, 6, and 12 *μ*g/mL) for 24 hours. Based on the time-course of p5 release by immunofluorescence assay (Figures 1(c) and 1(f)), conditioned medium (CM) from p5-primed hADMSCs collected on Day 1 was tested on BAECs. The CM from 12 *μ*g/mL p5-primed hADMSCs inhibited the cleavage of p35 and the CDK5 phosphorylation and more importantly suppressed phospho-p53 expression after Ca^2+^ ionophore and CaCl_2_ treatment (Figure 4(a), lane 10). Similarly to p5 alone, CM of p5-primed hADMSCs did not affect the phosphorylation of ERK1/2 induced by Ca^2+^ ionophore and CaCl_2_ treatment.

### 3.5. p5-Primed hADMSCs Protected Endothelial Cells from Calpain-Induced Toxicity

**Figure 5:**
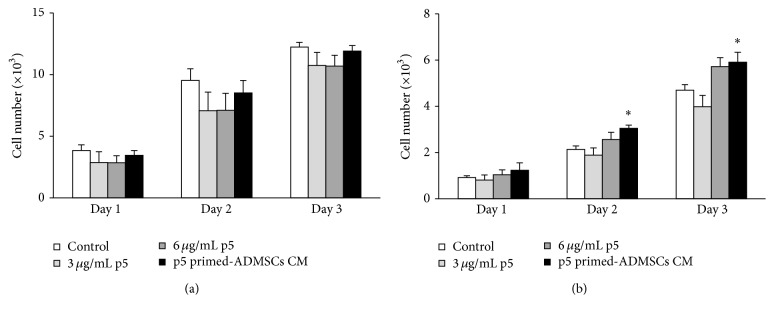
p5 and conditioned medium (CM) from p5-primed hADMSCs protected BAECs from calpain-induced cytotoxicity. The cultured BAECs were treated with p5 or CM from 12 *μ*g/mL p5-primed hADMSCs collected on Day 1 for 1 hour and then further treated with 5 *μ*M calcium ionophore A23187 and 2.5 mM CaCl_2_ for 10 minutes (a) or 2 hours (b). The cell proliferation was monitored for 3 consecutive days using the alamarBlue assay. Data are means ± SD (error bars). Significance (denoted as *∗*) was calculated compared to the control using Student's *t*-test (with *p* < 0.05, *n* = 3).

As the Ca^2+^ ionophore and CaCl_2_ treatment activated apoptosis related genes, such as p53 and caspase-3, we further examined the cytotoxic effects of the combined treatment of Ca^2+^ ionophore and CaCl_2_ towards BAECs. Compared to the untreated cells, the 10 minutes' treatment of Ca^2+^ ionophore and CaCl_2_ significantly inhibited BAECs proliferation by 60.9% (*p* < 0.01) whereas 2 hours' treatment inhibited BAECs growth by 90.5% (*p* < 0.01) in a 24-hour alamarBlue assay. We then investigated whether p5 or p5-primed hADMSCs would protect BACEs against the cytotoxicity induced by Ca^2+^ ionophore and CaCl_2_ treatment. Two concentrations of p5 (3 and 6 *μ*g/mL) and conditioned medium (CM) from 12 *μ*g/mL p5-primed hADMSCs collected on Day 1 were tested on BAECs. The proliferation assays showed that p5 and CM of p5-primed hADMSCs did not affect the BAECs numbers with the 10 minutes' treatment of Ca^2+^ ionophore and CaCl_2_ through 3 days (Figure 5(a)). However, compared with the 2 hours' treatment of Ca^2+^ ionophore and CaCl_2_ alone, the BAECs numbers in 6 *μ*g/mL p5 group increased by 13% on Day 2, 20% on Day 3, and 22% on Day 4 although these increases were not statistically significant (Figure 5(b)). More encouragingly, the BAECs numbers in p5-primed hADMSCs CM group increased by 35% on Day 2, 43% on Day 3 (*p* < 0.05), and 26% on Day 4 (*p* < 0.05) compared with the 2 hours' treatment of Ca^2+^ ionophore and CaCl_2_ alone (Figure 5(b)). The coculture of p5-primed hADMSCs and BAECs warrants the further evaluation of the p5-primed hADMSCs protection against BAECs cytotoxicity induced by the calpain activation.

## 4. Discussion

In the present study, our results demonstrated that, by simply incubating hADMSCs with p5, hADMSCs can uptake p5 and release biologically functional p5 in a time-dependent kinetics as well as in the dosage sufficient to inhibit the cleavage of p35, the phosphorylation of CDK5, and the upregulation of p53 induced by the activation of calpain in endothelial cells. As such, p5-loaded hADMSCs might be employed to protect endothelial cells from CDK5-p25 hyperactivity-induced toxicity caused by the intracellular Ca^2+^ rise and calpain activation under the exposure to stress, such as hypoxia/ischemia injury, oxidative stress, and inflammation.

The use of MSCs as an active targeted-delivery vehicle for therapeutic agents is especially attractive because MCSs have proven to be permissive to small compound incorporation, immunoprotective and pathotropic. Considering the limiting number of MSCs which can be delivered* in vivo*, it is important to have enough drug incorporated into the MSCs in order to achieve a therapeutic drug concentration in the targeted tissues [18]. Numerous studies on the uptake of nanoparticles in hADMSCs have shown that the internalization of nanoparticles can be improved by modification of the nanoparticles, size control, proper incubation time, and nanoparticle concentration [19]. In the current study, we aimed to load MSCs with CDK5 inhibitory peptides, the molecules being much bigger than nanoparticles, to inhibit the CDK5-p25 activity. The large peptides may be problematic with regard to the incorporation of peptides into the cells, which is controlled by both endocytosis into and exocytosis out of the cells. We therefore chose the smallest available truncated peptides of p35. p5 is a 24-residue mimetic peptide of p35 C-terminal and more readily diffusible peptide that specifically inhibits CDK5-p25 activity but does not affect endogenous CDK5-p35 activity [12–15]. Our work demonstrated for the first time that, through a simple* in vitro* process of priming, hADMSCs incorporated a sufficient amount of p5.

To be effective, a therapeutic agent must first be stably loaded inside the cell carrier and then be released into the extracellular space slowly and steadily, instead of a quick release into the systemic circulation before the cells reach the target site, thereby giving the cell carrier time to migrate towards the stroke regions and reach as many target cells as possible after the systemic delivery. Our results showed that the uptake of p5 in hADMSCs increased in a time-dependent manner, starting at 1-hour incubation and gradually enriched in cytoplasm at the end of 24-hour priming. After internalization, p5-primed hADMSCs continuously released p5 into medium for 3 days, with the peak release within the first 48 hours, whereas the substantial amount of p5 remained in cytoplasm for more than 5 days. The incorporation and release kinetics of nanoparticles in MSCs for the treatment of malignant glioma revealed that nanoparticles demonstrated an initial burst release from cells within the first 4 hours followed by a steady release over 9 days [20]. This kinetics is particularly advantageous since priming MSCs with p5 requires at least 8 hours for the maximum loading, during which the burst release of p5 is complete and therefore the p5 loaded in the cells are in the sustained release phase. As such, p5 retained within the cells act as intracellular drug depots, gradually releasing the encapsulated peptide. The timed drug release can be further designed by the modification of “internal” and “external” triggers. A pH-sensitive linker releases drugs upon nanoparticle along a pH gradient [21]. Considering that the hypoxic regions usually result in a low pH microenvironment, pH-dependent release could allow controlled drug release only in the targeted stroke sites. Taken together, although highly promising, a better understanding of drug release from MSCs may offer the new means of more specific, selective, timed, and controlled release of drugs* in vivo*.

It was also critical to ensure that drug loading did not affect the viability of MSCs or their native properties, especially their pathotropic properties. Several studies proved that loading anticancer-drug nanoparticles or even chemotherapeutic drugs did not affect the short-term or long-term viability of MSCs [19, 22]. Our work also confirmed that neither chemotherapeutic drug paclitaxel nor p5 loading affects the viability of MSCs (unpublished data). A property critical to the use of MSCs as drug carriers is their migratory potential.* In vitro* transwell experiments have demonstrated that nanoparticles loaded MSCs still possess the migration capacity towards chemoattractant and tumour cells (e.g., U87MG and U251 glioma cells) [20, 23]. Similarly, our unpublished data showed that the uptake of cytotoxic drug doxorubicin did not impair the migratory properties of MSCs to lung cancer cells.

CDK5 is a serine/threonine kinase and plays pivotal roles mostly in neuronal development and survival [1]. It is selectively activated in neurons by its noncyclin activators p35 and p39. The increasing evidences suggest that CDK5 becomes a cell death mediator when its activator p35 is cleaved to p25 by calpain [7, 8]. This inappropriate activation of CDK5 has been reported in neuronal death involved in the pathophysiology of several neurodegenerative diseases, including Alzheimer's disease, amyotrophic lateral sclerosis, Parkinson's disease, and prion-related encephalopathies. In addition, recent studies show that overactivation of CDK5 is a crucially prodeath signal in stroke [1]. The accumulation of p25 after transient forebrain ischemia activated CDK5 and induces CA1 cell death [24]. Hypoxia/ischemia injury in neonatal rats also caused CDK5 activation and increased neuronal apoptosis [12]. So far more than 20 specific substrates of CDK5 have been identified, including a number of substrates linked to apoptosis, such as Tau, p53, caspase-3, Bax, and Bcl-2 [1, 25]. p5 was reported to specifically inhibit the CDK5-p25 signaling pathway [12–14]. Furthermore, p5 reduced A*β*-induced Tau hyperphosphorylation and apoptosis in cortical neurons and decreased cleaved caspase-3 levels and neuronal apoptosis in Alzheimer's disease mouse model and a rat hypoxia/ischemia injury model [12–15].

In the present study, we examined whether p5 and p5-primed MSC inhibited CDK5 activity and reduced the markers of apoptosis induced by calpain in endothelial cells. The western blotting results showed that Ca^2+^ ionophore combined with CaCl_2_ treatment increased the cleavage of p35, the phosphorylation levels of CDK5 and ERK1/2 transiently, and p53 expression gradually at 10 minutes' treatment whereas caspase-3 was activated at 4 hours of treatment, much later than p53. More importantly, both p5 alone and p5-primed hADMSCs suppressed the cleavage of p35, the phosphorylation of CDK5, and the upregulation of p53 induced by calpain, suggesting that p5-loaded hADMSCs may protect cells from apoptosis via inhibiting p53. p53 is a critical modulator of cellular stress responses and is activated through diverse mechanisms. It was reported that CDK5 interacted with p53 and increased its stability by preventing HMD2-induced p53 ubiquitylation and downregulation, leading to accumulation of p53 in neurons [25]. CDK5 may also facilitate the function of p53 and p73 transcriptional complexes [26]. Whether CDK5 regulates p53 transcriptionally, translationally, or posttranslationally in endothelial cells needs to be further investigated. Besides, the previous studies showed that CDK5 protected neurons from cell death by direct interaction and activation of the antiapoptotic protein Bcl-2 and in PC12 neuronal cells via inhibition of sustained activation of ERK1/2 [1]. However, pERK1/2 levels were not affected by the p5 inhibition of CDK5 in this study, suggesting that ERK1/2 was activated in endothelial cells by Ca^2+^ ionophore and CaCl_2_ treatment independently of CDK5 signaling. Since the* in vivo* p5 inhibition studies reduced active caspase-3 levels at 24 hours after hypoxia/ischemia injury and 10 days in Alzheimer's disease mice, future work will be carried out to assess the long-term protective effects of p5-primed hADMSCs [12, 14].

## 5. Conclusions

To our knowledge, this is the first demonstration that, without any genetic manipulation, mesenchymal stem cells can be loaded* in vitro* with small bioactive peptides blocking CDK5 hyperactivity. The potential of use for p5-loaded hADMSCs in stroke and regulation of cell growth in other disease models such as cancer needs to be explored and their therapeutic efficacy may be further investigated in animal and human clinical trials.
